# Immunological insights into the occurrence of *Lawsonia intracellularis* in horses from southern Brazil using flow cytometry

**DOI:** 10.14202/vetworld.2025.755-762

**Published:** 2025-04-07

**Authors:** Yasmin Ampese Matté, Débora Zini Baldasso, Mariana Antunes Rezende, Jean Francisco Maldaner Lui, Ana Clara Seibel, João Antônio Guizzo, Rafael Frandoloso, Luiz Carlos Kreutz

**Affiliations:** 1 Graduate Program in Bioexperimentation, Laboratory of Advanced Microbiology and Immunology, School of Agricultural Sciences, Innovation and Business, University of Passo Fundo, 99052-900 Passo Fundo, RS, Brazil; 2 AFK Imunotech, Passo Fundo, RS, Brazil

**Keywords:** antibody test, Brazil, diagnosis, equine proliferative enteropathy, flow cytometry, *Lawsonia intracellularis*, vaccine

## Abstract

**Background and Aim:**

*Lawsonia intracellularis* is an obligatory intracellular bacterium associated with equine proliferative enteropathy (EPE), which significantly impacts equine health. Despite its clinical relevance, epidemiological and diagnostic approaches for this infection in horses have remained underexplored. This study aimed to evaluate the humoral immune response in horses immunized with an experimental vaccine for *L. intracellularis* and to determine the occurrence of anti-*L. intracellularis* antibodies in horses from southern Brazil using the flow cytometry antibody test (FCAT).

**Materials and Methods:**

A total of 12 horses were immunized with an experimental vaccine containing inactivated *L. intracellularis* and adjuvants. Blood samples were collected on day 0 (D0) and every 7 days until day 35 (D35) to monitor the antibody response using FCAT. In addition, serum samples from 79 horses from the North and Northwest regions of Rio Grande do Sul were tested to determine the occurrence of anti-*L. intracellularis* antibodies. The FCAT protocol was optimized for equine samples, and a cut-off fluorescence threshold was determined using Receiver Operating Characteristic curve analysis.

**Results:**

FCAT demonstrated high accuracy, with a sensitivity of 100% and specificity of 92% at a fluorescence cut-off of ≥15%. Immunization triggered a robust humoral response, with a significant increase in fluorescence from day 7 to day 35. In the occurrence analysis, 26% of the horses tested positive for anti-*L. intracellularis* antibodies. The highest occurrence was observed in females (35.8%) and horses aged 16–22 years (50%).

**Conclusion:**

This study provided evidence that *L. intracellularis* infection is relatively common among horses in southern Brazil. FCAT was a sensitive and specific diagnostic tool for detecting anti-*L. intracellularis* antibodies in horses. The findings highlight the need for better diagnostic and preventive measures to control EPE in equine populations.

## INTRODUCTION

*Lawsonia intracellularis* is an obligate intracellular bacterium responsible for equine proliferative enteropathy (EPE), a disease affecting the enterocytes of the ileum and the proximal portion of the cecum [1, 2]. This condition predominantly affects foals aged 3–7 months who present with clinical signs such as lethargy, anorexia, diarrhea, colic, weight loss, and fever [3–5]. Although it is primarily observed in foals, *L. intracellularis* can also affect adult animals, producing similar symptoms [6]. In addition to horses, *L. intracellularis* also causes clinical disease in other species, including pigs, rabbits, and hamsters [7–10].

The diagnosis of gastrointestinal infections in horses, including those caused by *L. intracellularis*, has historically been overlooked, resulting in a limited understanding of its prevalence in equine populations. Reports of *L. intracellularis* infection in horses have emerged from multiple regions, including Canada [2], United States [11], Switzerland [12], England [13], Belgium [14], Israel [15], Japan [16], and Germany [17]. In Brazil, confirmed cases have been reported in Minas Gerais [18], Rio de Janeiro [19], Paraná [20], and Rio Grande do Sul [21]. However, due to the subclinical nature of many infections and the limited scope of epidemiological studies, the true prevalence of *L. intracellularis* in horses may be underestimated or influenced by sampling bias. Although the full impact of this infection on horses remains unclear, diarrhea-related foal losses occur frequently in stud farms and represent a significant cause of morbidity and mortality [22–24].

*L. intracellularis* infections can be diagnosed using a combination of molecular and serological methods. Real-time quantitative polymerase chain reaction is utilized to detect bacteria in stool samples or rectal swabs [25, 26], whereas indirect immunofluorescence antibody test and immunoperoxidase monolayer assay (IPMA) remain the most widely used assays for identifying anti-*L. intracellularis* antibodies in serum samples [27, 28]. Combining these methods improves diagnostic accuracy and reduces the likelihood of erroneous results. Serological testing is especially valuable for identifying past infections, supporting the development of seroepidemiological studies. However, the diagnosis of EPE remains underdeveloped, and the immune response to *L. intracellularis* in horses is poorly understood, complicating the interpretation of serological findings.

Recently, our research group introduced the flow cytometry antibody test (FCAT) for detecting anti*-L. intracellularis* antibodies in pigs. This method demonstrated superior sensitivity and specificity compared to conventional assays such as enzyme-linked immunosorbent assay and IPMA [29]. FCAT leverages live-attenuated bacteria from a commercial vaccine, offering enhanced diagnostic accuracy and faster execution times relative to existing techniques.

Despite the recognized clinical and economic impact of *L. intracellularis* on equine health, there remains a significant gap in the epidemiological and immunological understanding of this infection in horses. Existing studies have primarily focused on diagnosing *L. intracellularis* in foals using conventional serological and molecular methods; however, these approaches often lack sensitivity or specificity, leading to potential underestimation of infection prevalence. Furthermore, limited data exist on the immunological response of horses following exposure or vaccination against *L. intracellularis*, particularly regarding the use of novel diagnostic tools such as the FCAT. While FCAT has been validated for use in pigs, its application in equine serology remains underexplored, necessitating further research to determine its diagnostic efficacy and potential role in seroepidemiological studies. In addition, the absence of a commercial vaccine for horses underscores the need for evaluating experimental vaccines to inform future preventive strategies. Addressing these gaps is essential to advancing diagnostic methodologies, improving disease surveillance, and implementing effective control measures for *L. intracellularis* in equine populations.

This study aimed to evaluate the humoral immune response elicited by an experimental vaccine against *L. intracellularis* in horses and to determine the occurrence of anti-*L. intracellularis* antibodies in the equine population of southern Brazil using the FCAT.

## MATERIALS AND METHODS

### Ethical approval

The immunization procedures, blood sample collection from horses, and utilization of equine serum samples stored at the Veterinary Diagnostic Laboratory of the Veterinary Hospital, University of Passo Fundo, were conducted in compliance with ethical standards and were approved by the Ethics Committee on the Use of Animals in Research at the University of Passo Fundo (CEUA No. 001/2023).

### Study period and location

Horse vaccination and blood sampling were carried out from February to May 2023 at a private horse farm in the municipality of Passo Fundo, Rio Grande do Sul State, Brazil.

### Antigen

In the flow cytometry assay, a vaccine strain of *L. intracellularis* (Enterisol® Ileitis, Boehringer Ingelheim, St. Joseph, Missouri, USA) was used as an antigen, according to the protocol previously described by Baldasso *et al*. [29].

### Serum samples

In this study, the absence of sera from horses definitively known to be infected or uninfected with *L. intracellularis* necessitated the production of antibodies through immunization. Because a commercial vaccine for *L. intracellularis* in horses is unavailable, we used Enterisol® Ileitis (Boehringer Ingelheim). For this purpose, the *L. intracellularis* vaccine was inactivated at a concentration of 5 × 10^8^ bacteria per 2 mL dose. The vaccine was formulated with either a 10% aluminum hydroxide (Sigma Aldrich, St. Louis, Missouri, USA) or a 10% Montanide Gel01 adjuvant (Seppic, Richmond, Virginia, USA).

The vaccine was administered intramuscularly to 12 adult horses. With one exception, all horses received two doses of the vaccine, spaced 21 days apart: six horses received the vaccine containing aluminum hydroxide adjuvant, and the other six received the vaccine containing Montanide Gel01 adjuvant. Blood samples were collected from all animals on the day of initial immunization (D0) and then every 7 days until day 35 (D35) to monitor the kinetics of the humoral immune response. One horse experienced an allergic reaction to the first dose and was excluded from further immunization.

**Figure 1:**
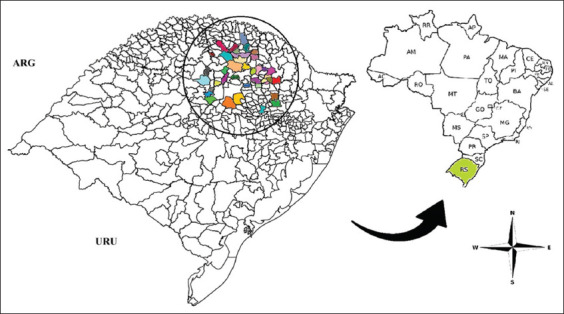
Geographic distribution of serum samples used to determine the occurrence of antibodies against *Lawsonia intracellularis* in horses (ARG=Argentina, URU=Uruguay). [Source: https://arquivofee.rs.gov.br/perfil-socioeconomico/estado/divisao-geopolitica-do-rs/].

Serum samples collected before immunization (D0) and after immunization (D35) were analyzed to estab- lish the cutoff value for the FCAT. In addition, a second panel comprising 79 equine serum samples collected between August and December 2022 from 39 municipalities in southern Brazil (Figure 1 and Table 1) was used to determine the occurrence of anti-*L. intracellularis* antibodies.

**Table 1:** Distribution of samples by municipality and respective quantities (n) used to determine the occurrence of anti-*Lawsonia intracellularis* in horses.

Municipality	n	Municipality	n	Municipality	n
Alto Alegre	2	Guabijú	2	Pontão	2
André da Rocha	2	Ibiraiaras	2	Quatro Irmãos	2
Barros Cassal	2	Ibirubá	2	Roca Sales	2
Bento Gonçalves	2	Ipiranga do Sul	2	Ronda Alta	2
Campos Borges.	2	Jacuizinho	2	Saldanha Marinho	2
Casca	2	Mato Castelhano	2	Santo Antonio do Planalto	2
Charrua	2	Muliterno	2	São Jorge	2
Ciríaco	2	Nicolau Vergueiro	2	Serafina Corrêa	2
Coxilha	2	Nova Alvorada	2	Sertão	2
Erechim	2	Nova Araçá	2	Tapejara	2
Fontoura Xavier	2	Nova Boa Vista	2	Tapera	2
Gentil	1	Paraí…	2	Veranópolis	2
Getúlio Vargas	2	Passo Fundo	5	Victor Graeff	1

#### Dilution of the primary antibody

Serum samples collected at D0 and D35 were evaluated at dilutions of 1:100 and 1:200 in phosphate-buffered saline (PBS, pH 7.4) with a final volume of 100 μL.

#### Secondary antibody dilution

To determine the optimal dilution of the secondary antibody (anti-horse immunoglobulin G [IgG] – fluorescein isothiocyanate [FITC], Sigma-Aldrich®), serum samples collected at D0 and D35 from the same horse were analyzed at a primary antibody dilution of 1:100. The secondary antibody was tested at dilutions of 1:1000, 1:2000, 1:2500, and 1:3000, each at a final volume of 100 μL. In addition, the percentage of immunofluorescence was evaluated after incubating the secondary antibody with *L. intracellularis* in the absence of the primary antibody to assess background fluorescence.

#### FCAT

The FCAT protocol follows a previously described methodology by Baldasso *et al*. [29]. A total of 10^6^ *L. intracellularis* bacteria were suspended in 100 μL of PBS and added to the wells of a 96-well polystyrene plate with conical bottoms. The plate was centrifuged at 3,000× *g* for 5 min, and after removing the supernatant, the bacterial pellet was resuspended in 100 μL of equine serum diluted to 1:100. The mixture was incubated at 37°C for 20 min. The plate was then centrifuged, and the bacteria were washed 3 times with 150 μL of PBS each (4,000 rpm, 5 min). Subsequently, 100 μL of PBS containing anti-horse IgG–FITC diluted at 1:2500 was added to each well and incubated at 37°C for 20 min. After a second centrifugation, the bacteria were washed 3 more times, resuspended in 300 μL of PBS, and transferred to 1.5 mL microtubes for analysis using a BD FACSVerse® cytometer (BD Biosciences, New Jersey, USA). A total of 100,000 events were acquired, and the bacterial population was identified as previously described by Baldasso *et al*. [29]. The results were expressed as the percentage of positive bacteria relative to the total bacterial population in the P1 region.

Serum samples collected at D0 and D35 were analyzed using the FCAT method. The results were used to establish the cutoff point for positivity using a receiver operating characteristic (ROC) curve, considering sensitivity and specificity values.

### Statistical analysis

All statistical analyses were performed using GraphPad Prism (GraphPad Software, San Diego, CA, USA). Data were evaluated for normality using the Shapiro–Wilk test. Descriptive statistics were expressed as means ± standard deviation (SD) for continuous variables and as percentages for categorical variables.

Comparisons of immunofluorescence percentages at different antibody dilutions were conducted using Student’s t-test or one-way analysis of variance (ANOVA), followed by Tukey’s *post hoc* test for multiple comparisons where applicable. For non-normally distributed data, the Mann–Whitney U test or Kruskal–Wallis test with Dunn’s multiple comparison test was used.

The optimal cutoff value for the FCAT was determined using ROC curve analysis, with sensitivity, specificity, and area under the curve (AUC) calculated. The confidence intervals (95%) were estimated to assess the precision of the sensitivity and specificity values.

The kinetics of the humoral immune response were analyzed by comparing immunofluorescence percentages across time points (D0 to D35) using repeated measures ANOVA, with *post hoc* pairwise comparisons performed using Bonferroni correction to identify significant differences.

To determine the occurrence of anti-*L. intracellularis* antibodies in field samples, descriptive statistics were calculated, and associations between categorical variables (e.g., age and sex) and antibody occurrence were analyzed using Chi-square or Fisher’s exact tests, as appropriate. A significant level of p < 0.05 was considered statistically significant for all tests.

## RESULTS

### Evaluation of the primary antibody dilution

**Figure 2:**
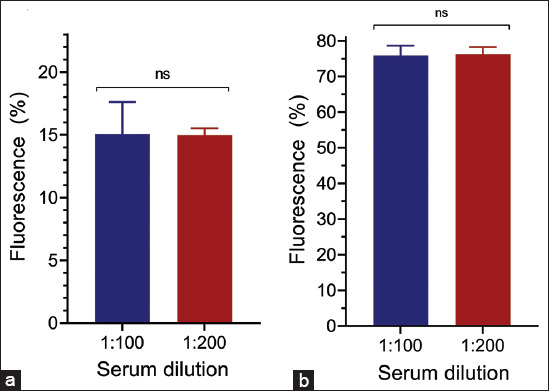
Identification of antibodies against *Lawsonia intracellularis* in horses by the FCAT. The serum samples were collected before vaccination at D0 (a) or at post-vaccination vaccination D35 (b) and tested at the indicated dilutions. The data were analyzed using student’s t-test and presented as the fluorescence mean ± standard error of the mean. ns=non-significant, FCAT=Flow cytometry antibody test.

Serum dilution (primary antibody) is an important parameter for diagnostic tests to maximize its sensitivity and specificity. This study is based on previous experience with the development of FCAT to test for anti-*L. intracellularis* antibodies in pigs [29], we analyzed only two serum dilutions: 1:100 and 1:200. On D0, we observed that the percentage of immunofluorescence associated with *L. intracellularis* was similar for both dilutions (Figure 2a). Similarly, for D35, there was no statistical difference in the percentage of immunofluorescence associated with *L. intracellularis* between the two dilutions (Figure 2b). In this context, for the other tests, we chose to use a 1:100 dilution of the primary serum to maintain the dilution standard defined for swine serum.

### Evaluation of the secondary antibody dilution

**Figure 3:**
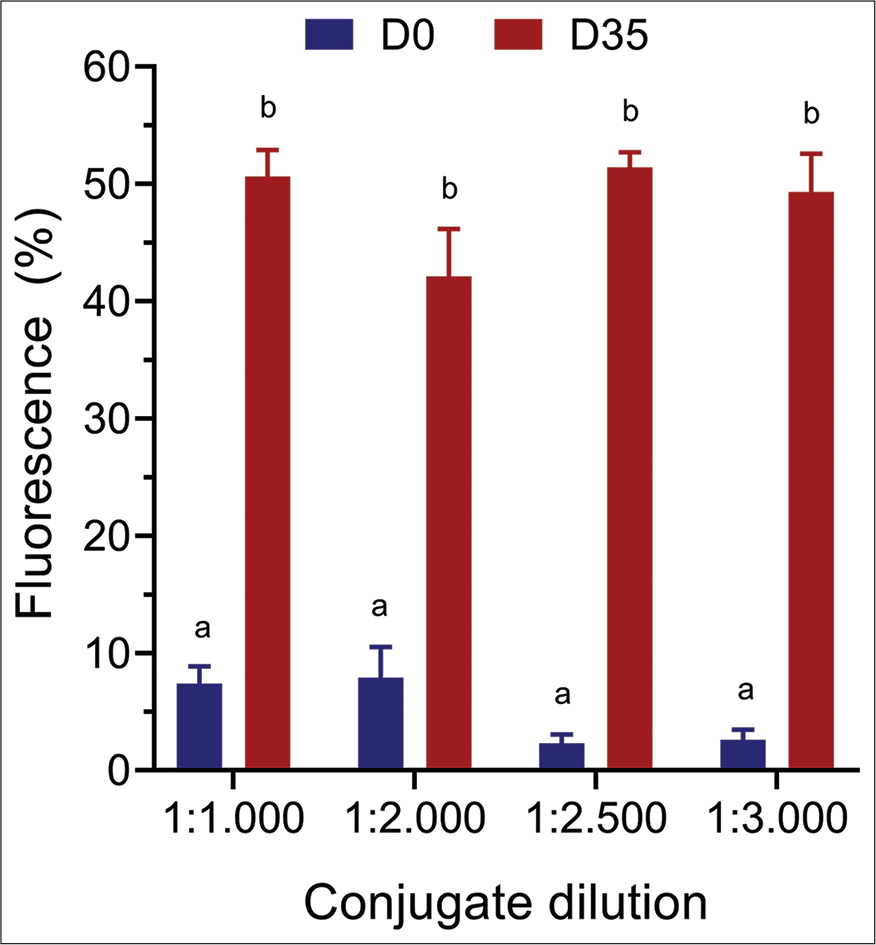
Effect of secondary FITC-conjugated antibody dilution on the percentage of fluorescence. The assay was performed using horse serum collected before (D0) or post-vaccination (D35) and FITC-labeled anti-horse conjugate at the indicated dilutions. The data are presented as the mean (±SEM) and were analyzed using one-way ANOVA (p < 0.05). SEM=Standard error of the mean, FITC=Fluorescein isothiocyanate, ANOVA=Analysis of variance.

After setting the primary antibody dilution at 1:100, we analyzed the influence of the secondary antibody dilution (anti-horse IgG – FITC) on the percentage of immunofluorescence associated with *L. intracellularis*. For this experiment, we used the equine sera of D0 and D35 at a dilution of 1:100 and the secondary antibody anti-horse IgG labeled with FITC at dilutions of 1:1000, 1:2000, 1:2500, and 1:3000. We observed that dilution of the secondary antibody at 1:2500 minimized false-positive results (Figure 3). We also analyzed the percentage of immunofluorescence associated with *L. intracellularis* in the absence of equine serum. The mean and standard error of the mean fluorescence obtained were 1:1000 = 1.6 ± 0.34, 1:2000 = 0.3 ± 0.03, 1:2500 = 0.32 ± 0.14, and 1:3000 = 0.07 ± 0.33. We observed no association between *L. intracellularis* and the secondary antibody. This result demonstrates that there is no reactivity with the secondary antibody, which could generate false-positive results.

### Cutoff point, sensitivity, and specificity (ROC curve)

**Figure 4:**
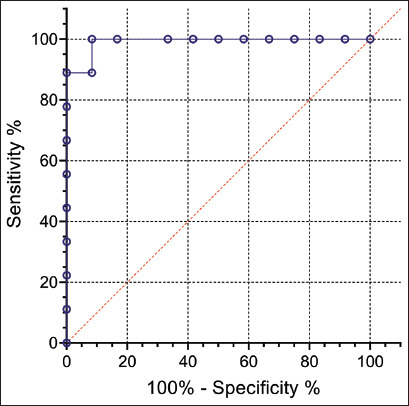
Receiver operating characteristics (ROC) obtained by analyzing horse serum samples collected at D0 (12 samples) and D35 (11 samples) using FCAT. The recommended cutoff point was ≥15%. In this cutoff value, the relative sensitivity and specificity were 100% and the specificity was 92.0%. The area under the ROC curve was 0.99. FCAT=Flow cytometry antibody test.

The percentage of immunofluorescence associated with *L. intracellularis* in the equine serum samples obtained at D0 and D35 were analyzed to verify the cutoff point with the best ability to discern between negative and positive samples. Although the number of samples used was limited (20 samples: 12 samples from D0 and 8 samples from D35), the analysis recommended a cut-off point ≥15% fluorescence to consider a positive sample. In this cutoff value, we obtained a relative sensitivity of 100% (95% confidence interval = 70.0%–100.0%) and specificity of 92.0% (95% confidence interval = 65.0%–100%). The ROC curve presented an AUC value of 0.99 (95% confidence interval = 0.96%–1.0%; p < 0.015), demonstrating a high level of accuracy for this assay (Figure 4). Thus, samples with a fluorescence percentage >15 were considered positive.

### Kinetics of immune response to immunization

**Figure 5:**
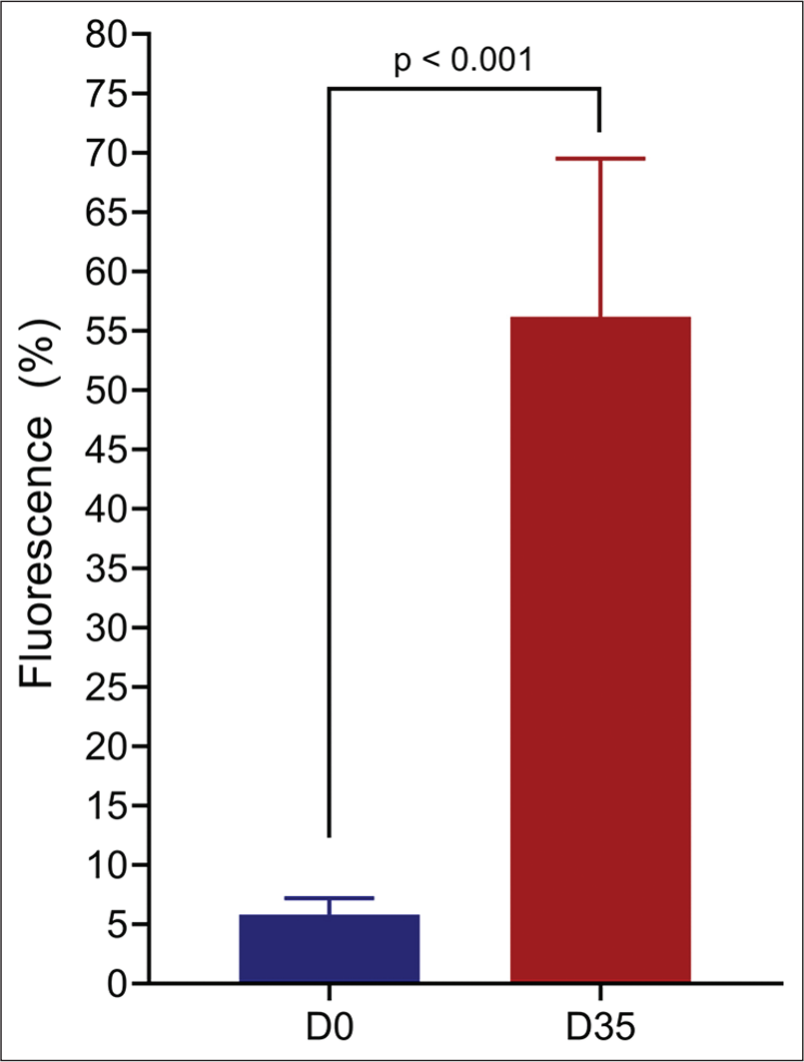
Anti-*Lawsonia intracellularis* antibody production in horses. The data represent the percentage of the mean fluorescence (±SEM) obtained by analyzing horse sera collected before (D0) or after vaccination (D35), with a cutoff point of ≥15% and were analyzed using Student’s t-test. SEM=Standard error of the mean.

After defining the value of the cutoff point capable of differentiating a positive from a negative serum sample, all samples from immunized horses, from D0 to D35, were analyzed to evaluate the kinetics of the immune response resulting from vaccination (Figure 5). The mean and SD of the groups were D0 = 5.83 ± 1.38, D7 = 38.2 ± 21.9, D14 = 52.7 ± 11.4, D21 = 48.6 ± 21.2, D28 = 54.0 ± 21.3, and D35 = 56.2 ± 13.3. We observed a considerable increase in fluorescence in D7 (1.007%) compared with D0, indicating that the vaccine-induced rapid and strong production of antibodies.

### Occurrence of antibodies in horses in southern Brazil

**Figure 6:**
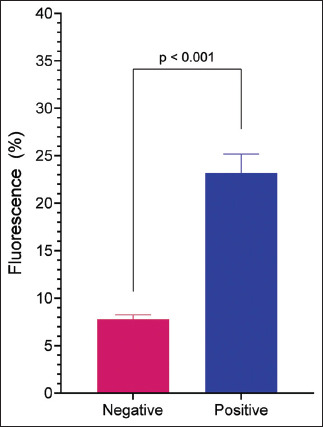
FCAT analysis of horse serum for the presence of *Lawsonia intracellularis* antibodies horse sera was collected from different municipalities in the northern and northeast regions of RS and analyzed using FCAT. The data represent the mean fluorescence (±SEM) and Student’s t-test was used (**** = p < 0.0001). FCAT=Flow cytometry antibody test, SEM=Standard error of the mean.

After standardization of the test, we analyzed 79 equine serum samples from 39 municipalities in the North and Northwest regions of Rio Grande do Sul to determine the occurrence of anti-*L. intracellularis* antibodies (Figure 1 and Table 1). The results are summarized in Table 2. Among the 79 samples, 21 (26%) were positive. The highest percentage of detection was found in the 16–22 age group (50%) and the lowest in the 11–15-year age group (14.2%). The highest percentage of detection was found in females (35.8%). The percentage of immunofluorescence in the field samples is shown in Figure 6.

**Table 2:** The occurrence of anti-*Lawsonia intracellularis* in horses according to age and sex in 39 municipalities in the northern and northwest regions of Rio Grande do Sul, Brazil.

Gender	0–2 years (%)	3–5 years (%)	6–10 years (%)	11–15 years (%)	16–22 years (%)	Total (%)
Female	1/3 (33.3)	3/7 (42.8)	6/20 (30)	2/6 (33.3)	2/3 (66.6)	14/39 (35.8)
Male	1/2 (50)	2/5 (40)	3/22 (13.6)	0/8 (0)	1/3 (33.3)	7/40 (17.5)
Total	2/5 (40)	5/12 (41.6)	9/42 (21.4)	2/14 (14.2)	3/6 (50)	21/79 (26,5)

The results are expressed as the number of positive samples (+) over the total sample number (n) and their respective percentage (%). Of the 79 tested samples, 21 were positive using the FCAT method. FCAT=Flow cytometry antibody test

## DISCUSSION

In this study, we demonstrated that the FCAT is a highly sensitive and specific method for diagnosing anti-*L. intracellularis* antibodies in horses and confirmed the presence of these antibodies in horses from the southern region of Brazil. In addition, we evaluated the immune response of horses immunized with an experimental *L. intracellularis* vaccine, demonstrating its efficacy in stimulating a specific immune response in horses.

The FCAT method was used to detect antibodies in horses. For horses, FCAT showed a sensitivity of 100% and specificity of 92%, compared to 89% and 100%, respectively, for the IPMA. This finding positions FCAT as a highly accurate diagnostic tool for detecting anti-*L. intracellularis* antibodies. Although initially developed for detecting anti-*L. intracellularis* antibodies in pigs, this study demonstrates its applicability to the diagnosis of horses. Alongside IPMA, which has already been established for equine diagnosis with high specificity [30], FCAT presents an innovative approach for the serological diagnosis of *L. intracellularis* in horses.

We also evaluated the humoral immune response of horses following intramuscular immunization with an investigational vaccine for *L. intracellularis*. FACT analysis showed low levels of fluorescence before immuniza-tion (D0), suggesting an absence of prior exposure to *L. intracellularis*. However, by day 35 (D35) after vaccination, there was a significant increase in the mean fluorescence (p < 0.001, Figure 5), indicating a rapid and robust immune response. A previous study by Pusterla *et al*. [31] on a live-attenuated vaccine administered intrarectally reported seroconversion in foals 21 days after the first dose, while another study observed no seroconversion 60-day post-immunization [32], likely due to variation in the vaccine dose. Our study demonstrates that vaccination effectively stimulates a specific immune response and induces a sustained immune response throughout the study period.

This study primarily focused on the humoral immune response triggered by vaccination. For a complete evaluation of the effectiveness of the vaccine, future research should evaluate additional parameters, such as protection against infection or reduction in the severity of clinical symptoms, to comprehensively assess vaccine efficacy.

After standardizing the FCAT method for horses, we evaluated the presence of anti-*L. intracellularis* antibodies in the equine population of Rio Grande do Sul. Among the 79 samples analyzed, 26% were positive for this bacterium, indicating that infection by this bacterium is relatively common in this region. The highest occurrence was observed in horses aged 16–22 years (50%), suggesting greater exposure to the pathogen over time. Females exhibited a higher seropositivity rate (35.8%) compared to males (17.5%). These findings imply that the animals were exposed to the bacteria because no commercial vaccines are available in Brazil. Potential sources of contamination include contact with other animals, such as pigs and rodents, and the movement of horses between farms for breeding purposes. These observations are consistent with previous findings in which females showed higher seropositivity rates [21, 33].

This study is not the first to demonstrate the seroprevalence of *L. intracellularis* in horses in Brazil. Previous research in Minas Gerais [18] and Paraná [20] identified low antibody prevalence, whereas a recent study in Rio Grande do Sul [21] reported a high preva- lence in Thoroughbred horses from stud farms with a history of diarrhea. In contrast, our study evaluated a broader equine population across the northern and northwest regions of the state, revealing a seroprevalence rate of 26%. These findings confirm that *L*. *intracellularis* infection is widespread in Brazil, particularly in Rio Grande do Sul, and emphasize the importance of including *L. intracellularis* in the differential diagnosis of equine enteric diseases. The presence of anti-*L. intracellularis* antibodies may indicate past or ongoing infections, potentially affecting animal health, therefore, implementing infection control measures, including management strategies and educating horse owners on prevention is crucial.

## CONCLUSION

This study highlights the utility of the FCAT as a highly sensitive and specific diagnostic tool for detecting anti-*L. intracellularis* antibodies in horses, achieving a sensitivity of 100% and specificity of 92%. Our results also demonstrated the efficacy of the experimental vaccine in eliciting a robust humoral immune response, with a significant increase in antibody levels observed by day 35 post-immunization. In addition, occurrence analysis revealed that 26% of horses in the North and Northwest regions of Rio Grande do Sul tested positive for *L. intracellularis* antibodies, with higher seropositivity rates observed in females and older horses. These findings confirm that *L. intracellularis* infection is relatively common in the region and underscore the need for improved diagnostic and preventive measures in equine populations.

One of the strengths of this study is the successful adaptation of FCAT, previously developed for pigs, for use in horses, offering an innovative, rapid, and reliable diagnostic alternative to traditional methods such as IPMA. Furthermore, the study provided valuable insights into the immune kinetics of horses vaccinated against *L. intracellularis*, demonstrating the vaccine’s ability to induce a strong and sustained immune response.

However, this study has limitations. The small sample size for the vaccination trial may restrict the generalizability of the results, and the absence of long-term follow-up data precludes conclusions about the vaccine’s protective efficacy against clinical disease. Moreover, although the occurrence analysis covered a broad geographic area, it lacked detailed data on potential confounding factors, such as management practices, environmental conditions, and co-infections, which may influence infection rates.

Future research should focus on evaluating the effectiveness of vaccines in preventing clinical symptoms and reducing disease severity. Longitudinal studies with larger sample sizes are needed to monitor the durability of immune responses and the vaccine’s protective efficacy under field conditions. In addition, investigations into the transmission dynamics of *L. intracellularis* and its potential reservoirs in mixed-species environments are essential for developing targeted control measures.

In conclusion, this study advances the understanding of *L. intracellularis* infection in horses and provides a foundation for improving diagnostics, vaccine development, and infection control strategies for equine health.

## AUTHORS’ CONTRIBUTIONS

LCK: Conceptualization. YAM, LCK, DZB, JAG, JFML, ACS, MAR, and RF: Methodology. YAM and LCK: Data curation, writing, and project administration. All authors have read and approved the final version of the manuscript.
